# Rheumatic heart disease in the South West region of Cameroon: a hospital based echocardiographic study

**DOI:** 10.1186/s13104-018-3341-6

**Published:** 2018-04-03

**Authors:** Clovis Nkoke, Anastase Dzudie, Christelle Makoge, Engelbert Bain Luchuo, Ahmadou Musa Jingi, Samuel Kingue

**Affiliations:** 1 Buea Regional Hospital, Buea, South West Region Cameroon; 2 Faculty of Medicine and Biomedical Sciences, University of Yaounde 1, Yaounde, Cameroon; 3 Douala General Hospital, Douala, Cameroon; 4 Athena Institute for Research on Innovation and Communication in Health and Life Sciences, Vrije Universiteit, Amsterdam, The Netherlands

**Keywords:** Rheumatic heart disease, South West region, Cameroon

## Abstract

**Objective:**

Rheumatic heart disease (RHD) prevails as a major public health problem in sub-Saharan Africa. In Cameroon, reports on RHD have been so far limited to a few cities. We sought to describe the demographic, clinical and echocardiographic features of rheumatic heart disease in the Buea Regional Hospital, South West region of Cameroon. Echocardiography reports between June 2016 and June 2017 were reviewed. The diagnosis of RHD was based on the World Heart Federation Criteria for the diagnosis of RHD.

**Results:**

A total of 669 echocardiograms were performed over the 1 year study period. Twenty-one (3.1%) had a definite echocardiographic diagnosis of RHD. There were 14 (66.7%) females. The age range was 13–94 years with a mean age of 47.8 ± 20.3 years. The most common indications for echocardiography were heart failure (47.6%), and dyspnea (42.9%). The mitral valve was the most commonly affected valve in 80.9% of cases. The most common valve lesion was isolated mitral stenosis (42.9%), followed by isolated mitral regurgitation (28.6%). There were no lesions on the tricuspid and pulmonic valves. Severe lesions were found in 80.9% of the patients. The complications were pulmonary hypertension (66.7%) and atrial fibrillation (9.5%).

## Introduction

Rheumatic heart disease (RHD) is a major public health problem in low and middle income countries where it continues to claim the lives of many children and young adults [1–4]. It is a leading cause of heart failure in children and young adults in sub-Saharan Africa [1, 5].

Rheumatic heart disease results from repeated episodes of acute rheumatic fever (ARF) following a group A streptococcal throat infection. There are about 15.6 million cases of ARF/RHD worldwide, with about 233,000 deaths yearly mostly in children and young adults in developing countries [2, 3]. Deaths from rheumatic heart disease results from complications such as heart failure, cardio-embolic stroke, atrial fibrillation, pulmonary hypertension, pregnancy related complications and complications related to surgery. Treatment of advanced rheumatic heart disease requires surgery which is inaccessible in resource limited settings. It is a disease that is preventable.

The prevalence of RHD varies from one country to another and even within the same country. In Nigeria, the prevalence has been shown to range from 3.7 to 38.5% [6–9], with some authors reporting a decline in the prevalence of RHD. This decline in RHD prevalence was attributed to improvements in the socio-economic condition and increased access to antibiotics for the treatment of sore throat [10]. In Cameroon, reports of Rheumatic heart disease have so far been limited to a few cities. We sought to describe for the first time the demographic and echocardiographic pattern of RHD in the South West region of Cameroon.

## Main text

### Methods

#### Study design and setting

This was a cross-sectional study conducted in the Buea Regional Hospital, South West region of Cameroon. The echocardiography register was surveyed from June 2016 to June 2017. All echocardiograms of patients with diagnosis of Rheumatic heart disease were reviewed. RHD diagnosis was based on the World Heart Federation (WHF) criteria for echocardiographic diagnosis of RHD. Briefly, RHD was defined by the presence of any evidence of mitral or aortic regurgitation seen in two planes associated with at least two of the following morphologic abnormalities of the regurgitating valve: restricted leaflet motility, focal or generalized valvular thickening, and abnormal sub-valvular thickening [11]. The echocardiographic reports were reviewed and used as the basis of RHD diagnosis. The echocardiography machine used was a Sonoscape S8^®^.

Only reports with sufficient information to make the diagnosis of RHD by the WHF criteria were included. Those with isolated functional regurgitating lesions were excluded from the analysis. Data were summarized by the type of valves involved, pathology (i.e., stenosis vs. regurgitation). Severity of valve lesions was described as mild, moderate, and severe according to American College of Cardiology/American Heart Association (ACC/AHA) guidelines [12]. Pulmonary hypertension was defined as a mean pulmonary artery pressure > 35 mmHg. Demographic and clinical data collected included age, sex and indication of echocardiography. The study was approved by the administrative authorities of the hospital acting as the local ethics committee. No formal ethics approval was required in this particular case.

#### Statistical analysis

Data were analyzed using SPSS version 22 for Windows. The results were presented as count and percentage for qualitative variables, and means and standard deviation for quantitative variables.

### Results

#### Demographic characteristics and indications of echocardiography

During the study period, a total of 669 echocardiographic examinations were performed. Twenty-one (3.1%) had a definite echocardiographic diagnosis of RHD. There were 16 (66.7%) females. The age range was 13–94 years with a mean age of 47.8 ± 20.3 years. There was no significant age difference between males and females (40.8 vs. 51.3 years, p = 0.28). The most common indications for echocardiography were heart failure (47.6%), and dyspnea (42.9%). Other indications for echocardiography were palpitations (4.8%) and atrial fibrillation (4.8%).

#### Pattern of valve lesions

The mitral valve was the most commonly affected valve in 80.9% of cases. The most common valve lesion was isolated mitral stenosis (42.9%) followed by isolated mitral regurgitation (28.6%) (Table 1). Mitral stenosis was more common in females (66.7%) than males but the difference was not significant (p = 1.00). Severe lesions were found in 80.9% of the patients. The complications were pulmonary stenosis (66.7%) and atrial fibrillation (9.5%).

**Table 1:** Distribution of valvular lesions

Valvular lesions	Frequency (%)
Mitral stenosis only	9 (42.9)
Mitral regurgitation only	6 (28.6)
Aortic stenosis only	1 (4.8)
Aortic regurgitation only	1 (4.8)
Mitral regurgitation + aortic regurgitation	1 (4.8)
Mitral stenosis + aortic regurgitation	1 (4.8)
Mitral regurgitation + mitral stenosis	1 (4.8)
Aortic regurgitation + aortic stenosis	1 (4.8)

### Discussion and conclusions

This is the first report on rheumatic heart disease from the South West region of Cameroon. In this study of echocardiography, we have shown that rheumatic disease affects 3.1% of patients referred for echocardiography. Heart failure and dyspnea were the most common indications of echocardiography. The mitral valve was the most commonly affected valve. The most frequent rheumatic valve lesion was isolated mitral stenosis followed by isolated mitral regurgitation. The tricuspid and pulmonic valves were not affected. More than two-third of rheumatic valve lesions were severe. The majority of the patients presented with complications.

A hospital based echocardiographic study in Yaounde covering a decade of activities showed that rheumatic heart disease affected about 6% of children aged ≤ 18 years referred for echocardiography [13]. The prevalence of 3.1% reported in our study is close to the 3.7% reported by Ogah et al. in Nigeria [6]. In the Nigerian Savannah, the prevalence of RHD was 9.8% in a hospital based echocardiographic study covering a period of 4 years [14]. The prevalence of RHD varies from one country to another and even within the same country. This can be due to differences in regions and mode of selection of patients. To the best of our knowledge, there is no community screening study of rheumatic heart disease in Cameroon. Echocardiographic screening studies of rheumatic heart disease in Africa have shown that the prevalence was 1.5% among school children in Uganda, 3% in Mozambique and 0.75% in Senegal [15–17]. The majority of patients in our study were referred for echocardiography following a clinical diagnosis of heart failure. Studies in Africa have shown that rheumatic heart disease is one of the dominant causes of heart failure. In the THESUS-HF registry of 1006 patients with heart failure from nine countries, RHD was the third most important cause of heart in adult Africans after hypertension and cardiomyopathies [5]. It is possible that the true prevalence of RHD in the community is higher as symptomatic patients are more likely to seek medical attention. Echocardiography has been shown to be superior to auscultation in the diagnosis of rheumatic heart disease. Widespread use in high prevalence settings is limited by cost and scarcity of skilled personnel.

The mitral valve was the most commonly affected valve in our study. This pattern of involvement is similar to that from other developing countries where the order of involvement is mitral followed by aortic, tricuspid and pulmonic valves [14]. This same pattern was reported in previous studies from other parts of Cameroon [13, 18]. Isolated mitral stenosis was the most common rheumatic lesion in our study (42.9%). This was different from reports from other parts of the country where the predominant rheumatic valve lesion was mitral regurgitation (49.2–59.7%) [13, 17]. The reason for this could not be ascertained. In Nigeria, Sani et al. also reported a predominance of mitral regurgitation in the Nigerian savannah [14]. Pulmonary hypertension was present in 66.7% of the patients in our study. This is in agreement with findings from local studies in Cameroon [13, 18]. About 10% of the patients presented with atrial fibrillation. Rheumatic heart disease is a major cause of atrial fibrillation in high endemic regions of the world. Rheumatic heart disease was the cause of atrial fibrillation in 25.6% of patients with atrial fibrillation in Yaounde [19]. The majority of the patients in our study presented with severe valvular lesions. The treatment of advanced rheumatic heart disease is usually surgical. But the cost of cardiac surgery usually makes this treatment procedure unaffordable to most families in resource limited settings like ours.

Our study has shown that rheumatic disease is still common in our setting with the majority of patients presenting with severe disease. There should be renewed emphasis on the prevention of this neglected disease.

## Limitations

This study was a hospital based review hence subject to bias. Our sample has potentially included patients with more symptomatic lesions since it was at a referral institution. Secondly, patients referred for echocardiography may be those with more severe rheumatic heart disease as patients with more severe lesions are more likely to seek medical attention. Despite this limitation, our study is the first to report on the pattern of rheumatic heart disease in this part of the country.

## Abbreviations

ACC: American College of Cardiology
AHA: American Heart Association
ARF: acute rheumatic fever
RHD: rheumatic heart disease
THESUS-HF: the sub-Saharan Africa survey of heart failure
WHF: World Heart Federation
